# The P-Type ATPase PA1429 Regulates Quorum-Sensing Systems and Bacterial Virulence

**DOI:** 10.3389/fmicb.2017.02449

**Published:** 2017-12-07

**Authors:** Yani Zhang, Jing Qin, Boren Tan, Weina Kong, Gukui Chen, Chao Zhang, Haihua Liang

**Affiliations:** ^1^Key Laboratory of Resources Biology and Biotechnology in Western China, Ministry of Education, College of Life Sciences, Northwest University, Xi’an, China; ^2^Western Reserve Academy, Hudson, OH, United States

**Keywords:** *Pseudomonas aeruginosa*, PA1429, quorum sensing, *Pseudomonas* quinolone signal, bacterial virulence

## Abstract

*Pseudomonas aeruginosa* is becoming an increasingly prevalent pathogen, capable of causing numerous life threatening infections in immunocompromised patients. The three hierarchically arranged quorum sensing (QS) systems, namely *las, rhl*, and *pqs* play key roles in coordinating virulence expression in *P. aeruginosa*. However, the regulatory mechanisms of the *pqs* system have not been fully elucidated. To identify new genes involved in synthesis of the *Pseudomonas* quinolone signal (PQS), a transposon mutagenesis library was constructed. PA1429 was found to inhibit PQS biosynthesis. The PA1429 deletion mutant also exhibited increased bacterial motility, biofilm formation, and virulence in a mouse model of acute lung infection. Interestingly, it also displayed reduced tolerance to oxidative stress. Mutated *pqsH* in the PA1429 deletion background restored bacterial susceptibility to H_2_O_2_. In addition, our data showed that PA1429 repressed the expression of *las* and *rhl* systems. Overall, these results provide new insights into the complex regulatory networks of quorum-sensing and virulence expression in *P. aeruginosa.*

## Introduction

*Pseudomonas aeruginosa* is an opportunistic human pathogen causing a wide range of infections in wounds, burns, and immunocompromised patients. It is also a major respiratory pathogen, which has a high impact on the morbidity and mortality of cystic fibrosis (CF) patients (Bjarnsholt et al., 2010). Due to its extraordinarily versatile metabolic abilities, varied virulence factors, high antibiotic resistance, and biofilm formation, *P. aeruginosa* is a formidable pathogen in the hospital environment (Breidenstein et al., 2011). It possesses multi-layered global regulatory networks that detect and react to endogenous and environmental molecules, triggering massive changes in genetic expression. Quorum sensing (QS) system is a central network that allows bacteria to coordinate gene expression in response to cell density, which has been estimated to control approximately 10% of the genes in *P. aeruginosa* (Lee and Zhang, 2015).

In *P. aeruginosa*, the three primary QS systems that have been identified are the *las, rhl*, and *Pseudomonas* quinolone signal (*pqs*) systems. The *lasI* and *rhlI* encode *N-*acyl-homoserine lactones (AHLs), *N*-3-oxo-dodecanoyl-homoserine lactone (3O-C12-HSL) and *N*-butyryl-homoserine lactone (C4-HSL), respectively. AHLs then bind and activate their regulators, LasR and RhlR, resulting in the regulation of target gene expression. Besides 3O-C12-HSL and C4-HSL, the *pqs* system is based on 2-heptyl-3-hydroxy-4-quinolone as a another signaling molecule (Passos da Silva et al., 2017). QS system is very complex and can be modulated by a range of positive and negative regulatory proteins. Noteworthy are the regulatory effects of Vfr, GacA, QscR, VqsR, RsaL, MvaT, Rpos, RpoN, and RsmA (Rampioni et al., 2006).

The *pqs* system is intertwined within the hierarchical system and plays a critical role in the pathogenicity of *P. aeruginosa.* Synthesis of PQS depends on the *pqsABCD* operon, which synthesizes the PQS precursor molecule 2-heptyl-4 (1H)-quinolone (HHQ). It is eventually converted into PQS by a *lasR* dependent monooxygenase, PqsH (D’Argenio et al., 2002; Gallagher et al., 2002). HHQ and PQS activate PqsR, a *lasR*-type transcriptional regulator PqsR, that enhance the biosynthesis of HHQ and PQS through activation of the *pqsABCDE* operons, forming a positive autoregulatory feedback loop for quinolone synthesis (Deziel et al., 2004). PQS regulates virulence factors such as elastase B, pyocyanin, hydrogen cyanide, and rhamnolipids (Gallagher et al., 2002; Diggle et al., 2003; Wade et al., 2005). Besides virulence factors, biofilm and membrane vesicle formation are under the control of PQS signaling (Diggle et al., 2007). However, the regulation of PQS has not been fully understood. In this study, a *P. aeruginosa* strain carrying the reporter *pqsH-lux* was subjected to random transposon mutagenesis. PA1429 was at first identified to be involved in PQS production. In addition, the deletion of PA1429 resulted in increased bacterial motility, biofilm formation, and virulence in a mouse model of acute lung infection. Our data clearly demonstrated that PA1429 is a new modulator of *P. aeruginosa* pathogenesis.

## Materials and Methords

### Strains, Growth Media, and Culture Conditions

The bacterial strains used in this study are listed in Supplementary Table S1. *P. aeruginosa* wild-type strain PAO1, its derivatives, and *Escherichia coli* were grown in Luria Bertani (LB) medium at 37°C. The following antibiotics were used. For *E. coli*: 15 μg/ml gentamicin (Gm), 50 μg/ml kanamycin (Kan), 100 μg/ml ampicillin (Amp), and 10 μg/ml tetracycline (Tet) in LB medium. For *P. aeruginosa*: 50 μg/ml Gm in LB medium or 150 μg/ml in *Pseudomonas* isolation agar (PIA), 100 μg/ml Tet in LB medium or 300 μg/ml in PIA, 300 μg/ml trimethoprim (Tmp), and 500 μg/ml carbenicillin (Cb) in LB medium.

### Gene Expression Assays

The plasmid pMS402 carrying a promoterless *luxCDABE* reporter gene cluster was used to construct promoter-*luxCDABE* reporter fusions as described previously (Duan et al., 2003). Promoter regions were amplified from chromosomal DNA by PCR using the primers designed based on PAO1 genome data^1^. They were cloned into the *Bam*HI-*Xho*I site upstream of the *lux* genes on the pMS402 plasmid. The primers used are listed in Supplementary Table S1. The resultant plasmids were transformed into *P. aeruginosa* by electroporation. Beside the plasmid-based reporter system, an integration plasmid CTX 6.1, originating from plasmid mini-CTX-*lux* was used to construct the chromosomal fusion reporter (Becher and Schweizer, 2000). The pMS402 fragment containing the MCS kanamycin-resistance marker, and the promoter-*lux* reporter cassette were then isolated and ligated to CTX 6.1, yielding CTX-*pqsH*-*lux*. The *P. aeruginosa* strain exhibiting reporter integration was obtained by electroporation. All constructs were sequenced to verify that no mutations had been incorporated.

The expression of *lux*-based reporters from bacteria grown in liquid culture were measured as counts per second (cps) of light produced in a Synergy 2 Plate Reader (BioTek), as described previously (Kong et al., 2015). Overnight incubated cultures of reporter strains were diluted to obtain an *A*_600_ of 0.2 and cultivated for an additional 2 h before use. Five microliters of fresh cultures were inoculated in parallel wells of a black 96-well plate with a transparent bottom containing 95 μl of medium with other components. A 60 μl volume of filter-sterilized mineral oil was added to prevent evaporation during the assay. Promoter activities were measured every 30 min for 20–24 h. Bacterial growth was monitored at the same time by measuring the OD at 600 nm in the Synergy 2 Plate Reader.

### Transposon Mutagenesis

The PAO1 strain containing the CTX-*pqsH*-*lux* reporter fusion gene was subjected to transposon mutagenesis using the mariner transposon vector pBT20 (Kulasekara et al., 2005). Briefly, the donor strain (*E. coli* SM10- *pir*) containing pBT20 and recipient PAO1 containing CTX-*pqsH*-*lux* were scraped from overnight plates and resuspended in 1 ml of M9 minimal medium. The donor and recipient strains were mixed together at a ratio of 2:1 and spotted on a dry LB agar plate, and incubated at 37°C for overnight. Mating mixtures were diluted and spread on PIA plates containing Gm at 150 μg/ml. A transposon mutant library was constructed by picking colonies grown in the selective plates. The mutants exhibiting altered expression of CTX-*pqsH*-*lux* were selected. The transposon insertion sites were determined by carrying out arbitrary primed PCR and subsequent sequencing of the PCR product (Friedman and Kolter, 2004).

### Construction of *P. aeruginosa* Mutants

For construction of gene knockout mutants, the previously described *sacB*-based strategy was employed (Hoang et al., 1998). To construct the PA1429 mutant, the upstream and downstream sequences flanking PA1429 were amplified from PAO1 genomic DNA. The upstream and downstream sequences were amplified using primer pairs 1429-up-s (*EcoR*I) and 1429-up-a (*BamH*I), and 1429-down–s (*BamH*I) and 1429-down-a (*Hin*dIII) (Supplementary Table S1), respectively. The two PCR products were digested and then cloned for gene replacement in the *EcoR*I/*Hind*III-digested suicide vector pEX18Ap carrying a *sacB* gene, yielding pEX18Ap-1429. Gene knockout mutants were obtained using the triparental mating procedure, in which the strain carrying the helper plasmid pRK2013 was used, along with the donor and recipient (Ditta et al., 1980). A similar strategy was used for generating the PA1429/*pqsH* deletion strain (ΔPA1429Δ*pqsH*). These resultant mutants were verified by PCR.

### Complementation of Mutants

To construct the complemented strain of ΔPA1429, the promoter and coding regions of PA1429 were integrated at the *attB* site in the ΔPA1429 mutant genome using mini-CTX1 system (Hoang et al., 2000). The DNA regions of the target genes were PCR-amplified using the primer pair 1429-up and 1429-down; these are listed in Supplementary Table S1. The resulting complemented mutant strain was verified by PCR analysis and named ΔPA1429/CTX-1429.

### PQS Assay

Quantification of PQS production was carried out as described previously (D’Argenio et al., 2002). Briefly, bacterial cultures incubated overnight were diluted 100-fold in fresh LB media and incubated at 37°C for 24 h. After incubation, 500 μl of culture was mixed with 1 ml of acidified ethyl acetate, vortexed vigorously for 2 min, and centrifuged at 16,000 *g* for 10 min. The upper organic layer was transferred to a fresh tube and allowed to dry overnight at room temperature. The following day, dried extracts were dissolved in 50 μl of a solution containing acidified ethyl acetate and acetonitrile mixed in a 1:1 proportion; Sample extracts were analyzed by thin-layer chromatography (TLC) analysis and quantitated by densitometry using ImageQuant software.

### Motility Assays

Motilities were assayed using varied media (Rashid and Kornberg, 2000; O’May and Tufenkji, 2011). Swimming media consisted of 0.3% agar supplemented with 1% tryptone and 0.5% NaCl. Swarming media was comprised of 0.5% agar supplemented with 0.8% Biotech nutrient broth and 0.5% glucose. Twitching media was LB broth with 1% agar. *P. aeruginosa* cultures were diluted to achieve an OD_600_ of 0.1, and 2 μl of the diluted bacteria was center-spotted on the surface of the agar plates. After the bacterial liquid was absorbed, swimming plates were incubated at 30°C for 14–16 h, twitching plates were incubated at 37°C for 24 h, while swarming plates were incubated at 37°C for 14–16 h. Photographs were taken with the Tanon 2500 imaging system.

### Biofilm Formation Assays

Biofilm formation assays were performed as previously described, with minor modifications (O’Toole and Kolter, 1998). Visualization of biofilm formation was done in 15 ml borosilicate tubes. Briefly, bacteria from overnight cultures were inoculated in LB medium supplemented with appropriate antibiotics at dilutions of 1:1000 and grown at 30°C for 22 h. Biofilms were stained with 0.1% crystal violet (CV) and tubes were washed with water to remove unbound dye. To quantify the biofilm on the borosilicate tubes, 800 μl of 95% ethanol was added to each well to elute the crystal violet. The absorbance of the pooled eluent was measured at 600 nm.

### H_2_O_2_ Sensitivity Assays

The H_2_O_2_ sensitivity disk assay was performed as follows. Briefly, bacterial strains were grown overnight in LB broth at 37°C, and cell concentration was standardized by adjusting the solution so that the optical density was 0.1 at 600 nm. Twenty-five milliliters of LB medium was poured into 100 mm Petri dishes after mixing with 250 μl of microbial cultures. Sterilized filter paper disks (6 mm in diameter) impregnated with 5 μl of 30% H_2_O_2_ was placed onto the surface of the medium. The plates were incubated overnight at 37°C for 24 h and the diameters of the zones of inhibition that developed were measured.

The assessment of tolerance exhibited by bacterial strains to H_2_O_2_ toxicity was performed as follows. Briefly, bacterial cultures incubated overnight were diluted to an approximate density of 10^6^ CFU/ml and treated with H_2_O_2_ (50 mmol/l) for 30 min at 37°C. Bacterial cells were centrifuged, washed thrice with PBS, and serially diluted in LB medium. Five microliters of bacterial suspensions were spotted on LB agar plates and incubated overnight at 37°C to determine viability.

### Mouse Model of Acute Pneumonia

All animal experiments were performed in strict accordance with the Regulations for the Administration of Affairs Concerning Experimental Animals approved by the State Council of People’s Republic of China (11-14-1988). All animal procedures were approved by the Institutional Animal Care and Use Committee (IACUC) of the college of life sciences of Northwest University with a permit number: NW-02-2014. Acute lung infection was performed as described previously (Li et al., 2014). Briefly, the stationary phases of bacterial strains grown in LB were harvested, washed three times with PBS, and serially diluted in PBS. For bacterial burden determination, 6-week-old male CD-1 mice (*n* = 11) were anesthetized with isoflurane and infected by nasal inhalation of a 50 μl bacterial suspension containing 2.5 × 10^7^ CFU of *P. aeruginos*a, infected mice (*n* = 6) were killed at 16 h, their lungs were aseptically removed, placed on ice, and subsequently homogenized in 1 ml cold PBS with a Dounce homogenizer. Homogenates then underwent serial dilution and plating on PIA. Colonies were counted after incubating for 24 h at 37°C.

### Statistical Analysis

All experiments were performed in triplicate and independently repeated three times. Data were analyzed using an unpaired Student’s *t*-test and ANOVA, and expressed as the mean ± SEM. The differences between means were considered to be significant (*p* < 0.05).

## Results

### Identification of Mutants with Altered *pqsH*-*lux* Expression

To identify new regulators affecting *pqsH* expression, a transposon insertion mutant library of *P. aeruginosa* PAO1 carrying the reporter *pqsH*-*lux* on its chromosome was constructed. Mutants with changed *pqsH* promoter activity were collected. In total, 11 mutants were selected. Arbitrary primed PCR and DNA sequencing determined the transposon insertion sites. The mutated genes identified are listed in **Table 1**.

**Table 1:** Mutants defective in *pqsH* expression.

Insertion site	Gene name or number	Description	Max fold
**Increased expression**			
649619	PA0590 (*apaH*)	Bis (5′-nucleosyl)-tetraphosphatase	2
3945216	PA3525 (*argG*)	Argininosuccinate synthase	3
4218710	PA3763(*purL*)	Phosphoribosylformylglycinamidine synthase	3
3767022	PA3356(*pauA*5)	Glutamylpolyamine synthetase	3
1557569	PA1429	Probable cation-transporting P-type ATPa	4
5450147	PA4854(*purH*)	Phosphoribosylaminoimidazolecarboxamide formyltransferase	2.5
**Decreased expression**			
813117	PA0745	Probable enoyl-CoA hydratase/isomerase	2
2450231	PA2228	Hypothetical protein	10
1557569	PA3011 (*topA*)	DNA topoisomerase I	3
1559485	PA1432 (*lasI*)	Autoinducer synthesis protein LasI	8
1549342	PA1423 (*bdlA*)	Cell wall/LPS/capsule	8

*Max fold, maximal ratio of expression between the mutant and the wild-type.*

Of the 11 transposon mutants, six mutants displayed increased *pqsH* expression, while the remaining five exhibited decreased *pqsH* expression. As expected, a known *pqs* system modulator of *lasI* was isolated along with a set of new loci that had not been described, such as PA1429, PA2228, and PA0745 (**Table 1**).

### PA1429 Represses the Expression of *pqsH*

**FIGURE 1:**
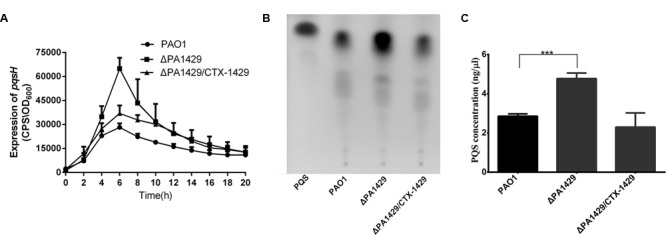
PA1429 represses the expression of *pqsH.* **(A)** The expression of *pqsH*-*lux* was measured in PAO1, ΔPA1429 mutant, and the complemented strain (ΔPA1429/CTX-1429). **(B)** PQS production was analyzed by TLC. PQS standard is shown on the first line. **(C)** Determination of PQS concentration in PAO1, ΔPA1429, and ΔPA1429/CTX-1429. Relative PQS concentration was determined by comparing PQS standard spots. Data are the averages of three independent experiments. Error bars represent means ± SEM. ^∗∗∗^*p* < 0.001, as determined by Student’s *t-*test.

Among the 11 transposon mutants, the PA1429 mutation resulted in significant increase in *pqsH* expression. PA1429 encodes a probable cation-transporting P-type ATPase. Since PA1429 had not been found to affect PQS production, it was essential to analyze it further. To verify that the mutation in PA1429 was responsible for the increased *pqsH* expression, a PA1429 mutant was constructed. The *pqsH*-*lux* reporter plasmid was transferred to the PA1429 mutant and PAO1 strains. Similar to the transposon mutant, the PA1429 mutant showed a higher *pqsH* expression than the wild-type strain (**Figure 1A**). Considering that *pqsH* is required for PQS synthesis, we next determined PQS levels in the PA1429 mutant by TLC and densitometry using ImageQuant software. As expected, the PA1429 mutant exhibited a more significant increase in the production of PQS than the wild-type PAO1. The ΔPA1429 complemented strain restored the level of PQS production to that observed in PAO1 (**Figures 1B,C**). These results indicate that PA1429 represses the expression of *pqsH*.

### The Expression of *pqsA, pqsR, las*, and *rhl* Were Enhanced in PA1429 Mutant

**FIGURE 2:**
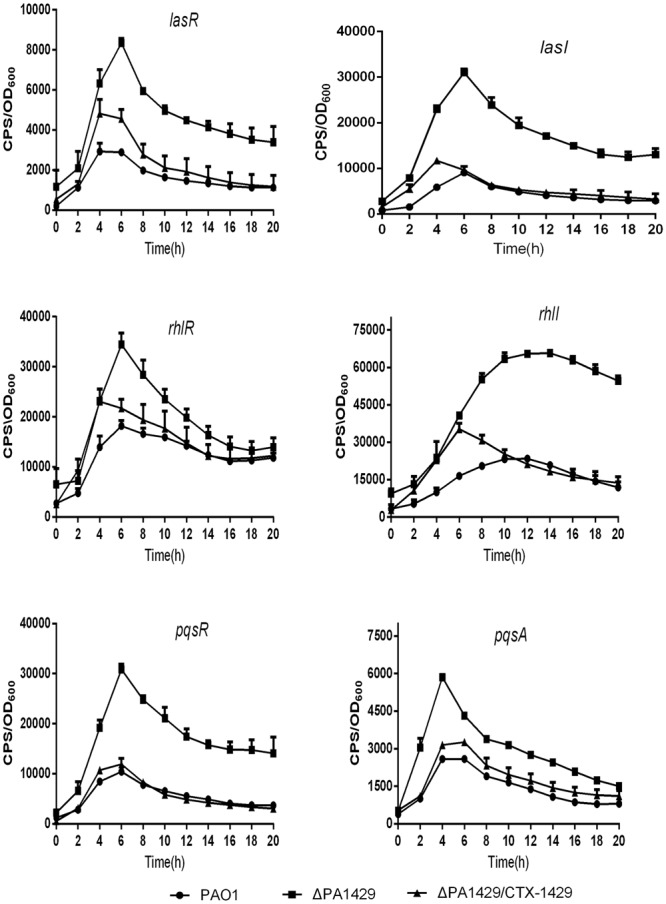
PA1429 represses the expression of *pqsR, pqsA, las, and rhl.* The expression of *pqsR*-*lux, pqsA*-*lux, lasR*-*lux, lasI*-*lux, rhlR-lux*, and *rhlI-lux* was measured in PAO1, ΔPA1429 mutant, and ΔPA1429/CTX-1429 strain. Data are the averages of three independent experiments. Error bars represent means ± SEM.

The major synthase genes and the precursor of PQS, named HHQ, are arranged in the *pqsABCDE* operon. This operon is under the control of *pqsR*, which activates the *pqsABCDE* operon while binding to PQS or HHQ, to create an autoregulatory loop (Lahiri et al., 2008; Heeb et al., 2011). Since the PA1429 mutant displays an increased extracellular PQS level, we postulated that it might also influence the expression of the *pqsA* operon and *pqsR*. As expected, the expression of *pqsR* and *pqsA* were increased in PA1429 mutant. After complemented the PA1429 mutant, the expression of *pqsR* and *pqsA* were almost decreased to the level of PAO1 (**Figure 2**). These results imply that a high level of PQS in PA1429 mutant activates *pqsR*, which in turn induces the expression of *pqsA*.

In *P. aeruginosa*, the *pqs* system is connected to both the *las* and *rhl* system. The *las* system upregulates *rhl, pqsABCDE, pqsR*, and *pqsH* (Williams and Camara, 2009), while *rhlR* negatively regulates *pqsR* transcription and inhibits PQS production, and PQS positively regulates *rhlR* (Wade et al., 2005; Wilder et al., 2011). We next sought to evaluate whether the expression of *las* and *rhl* systems could be affected in the PA1429 mutant. Therefore, the *lasR*-*lux, lasI*-*lux, rhlR*-*lux*, and *rhlI*-*lux* reporter plasmids were transferred to the PA1429 mutant and PAO1. The expression level was monitored during whole growth phase. Interestingly, the results showed that the expression of *lasR, lasI, rhlR*, and *rhlI* were increased in the PA1429 mutant compared with the PAO1 strain (**Figure 2**), indicating that PA1429 may play a role in the regulation of the *las* and *rhl* QS systems.

### PA1429 Mutant Exhibited Increased Motility and Biofilm Formation

**FIGURE 3:**
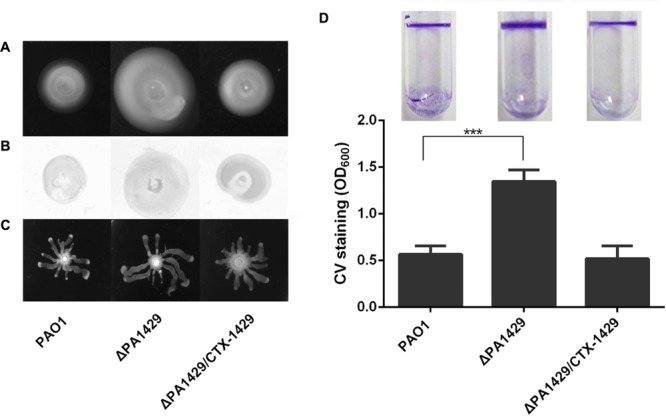
PA1429 affects motility and biofilm formation in *Pseudomonas aeruginosa*. **(A)** Swimming; **(B)** twiching; **(C)** swarming; **(D)** biofilm formation and Quantification of crystal violet staining of biofilms. Data are the averages of three independent experiments. Error bars represent means ± SEM. ^∗∗∗^*p* < 0.001, as determined by Student’s *t*-test.

The motility and biofilm formation abilities are major characteristics of bacterial pathogens and are strongly associated with virulence. Motility enables bacteria to escape hostile environments and move to safer living conditions. Biofilm formation during maturation could be affected by swarming motility (Shrout et al., 2006; Pamp and Tolker-Nielsen, 2007; Chow et al., 2011). To further define the function of PA1429, its effects on motility and biofilm formation were investigated. Deletion of PA1429 enhances swarming, swimming and twitching motility than the parental wild-type strain PAO1. The genetically complemented strain showed these phenotypes similar to those observed in the wild-type strain (**Figure 3**). We also examined whether PA1429 affected biofilm production. As shown in **Figure 3D**, the PA1429 mutant displayed hyper-biofilm phenotype when compared to the wild-type PAO1 and the complemented strain. These results clearly suggest that PA1429 is required for *P. aeruginosa* virulence expression.

### PA1429 Mutant Exhibited an Increased Sensitivity against Hydrogen Peroxide

**FIGURE 4:**
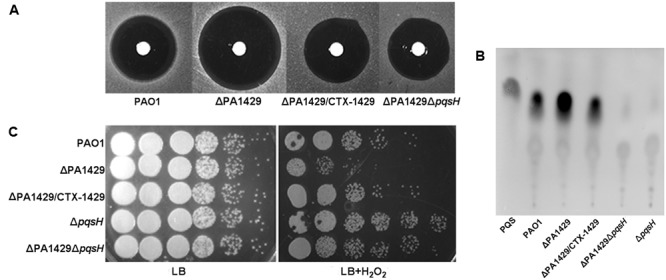
PA1429 mutant exhibited an increased sensitivity against hydrogen peroxide. **(A)** Growth inhibition by H_2_O_2_ as determined by agar diffusion assays. Bacteria were grown in LB meidium to stationary phase, and then inculated in LB soft agar (0.7%). Sterile filter disks were laid on top, and the disks were spotted with 5 μl 30% H_2_O_2_. **(B)** PQS production was analyzed by TLC. PQS standards are shown on the first line. **(C)** The tolerance exhibited by bacteria strain to H_2_O_2_ toxicity. After growth to stationary phase in LB meidium, cells were exposed to 0.03% H_2_O_2_ for 30 min, followed by detemination of CFU.

Previous studies have demonstrated that a high level of PQS increases bacterial sensitivity to stressful conditions (Bredenbruch et al., 2006; Haussler and Becker, 2008). Our data revealed that PA1429 negatively controls the PQS production, which led us to measure the sensitivity of PA1429 mutant toward hydrogen peroxide. The results showed that the PA1429 mutant exhibited a greater sensitivity toward hydrogen peroxide than wild-type PAO1. The complementation of the PA1429 mutant eliminated this sensitivity (**Figure 4A**). To verify that the high level of PQS in PA1429 was responsible for its phenotype, we constructed a double mutant, ΔPA1429Δ*pqsH*. The production of PQS was analyzed in ΔPA1429Δ*pqsH*. ΔPA1429Δ*pqsH* could significantly decrease the higher PQS production observed in the PA1429 mutant (**Figure 4B**). Further experiments showed that the sensitivity of the ΔPA1429Δ*pqsH* mutant against hydrogen peroxide was also abolished because of the absence of PQS (**Figure 4C**). Taken together, these results indicate that a high level of PQS in the PA1429 mutant strain make it more sensitive to oxidative stress.

### PA1429 Mutant Showed Increased Virulence in a Mouse Model of Acute Pneumonia

**FIGURE 5:**
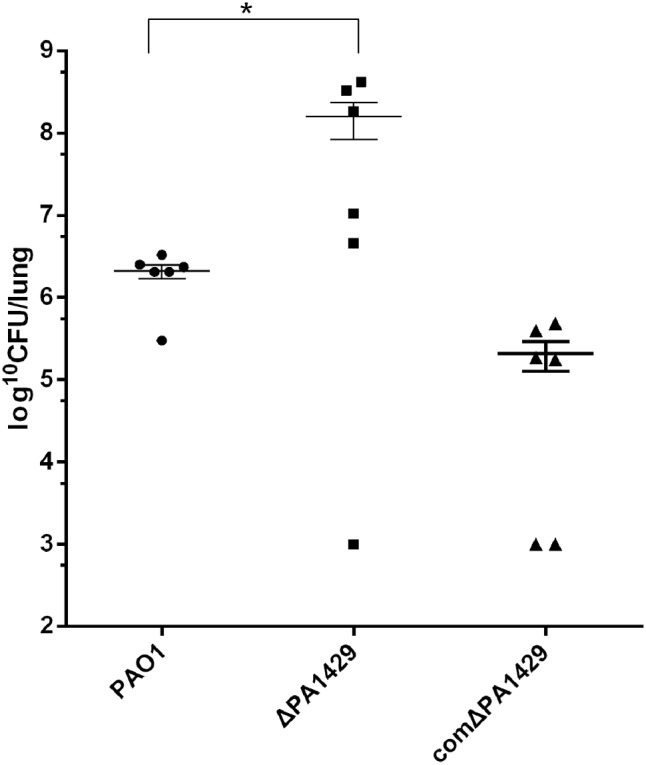
The lack of PA1429 leads to increased virulence of *P. aeruginosa* in acute pneumonia infection. Bacterial CFU in CD-1 mouse lungs (*n* = 6) after challenged with *P. aeruginosa*. Data represent average cfu/lung ± SEM. ^∗^*p* < 0.01, as determined by Student’s *t*-test when compared PAO1 against ΔPA1429.

The aforementioned results demonstrate that PA1429 mutant produces more PQS and has increased bacteria motility and biofilm formation. The pathogenesis of *P. aeruginosa* and its ability to colonize environments are strongly associated with motility (Yeung et al., 2009; Gellatly and Hancock, 2013). PQS is involved in the regulation of virulence factor production via PqsR. We further investigated whether the PA1429 mutant exhibited increased infection and systemic dissemination. We compared the bacterial burden on mouse lungs after 16 h infection with PAO1 and PA1429 mutant strains. As shown in **Figure 5**, the number of bacteria in the PA1429 mutant-infected lungs had a median value of 3.669 × 10^8^ CFU/lung. In contrast, the average bacterial burden in mice infected with PAO1 was approximately 100-fold lower, at 2.726 × 10^6^ CFU/lung. These results indicate that the PA1429 mutant could increase lung injury and inflammation.

## Discussion

In *P. aeruginosa*, the *pqs* system as the third QS regulatory network, controls the expression of numerous virulence factors (Kariminik et al., 2017). However, the mode of action of this system in most cases is unknown. We have a desire to learn more about the regulation of PQS production, with a specific focus on the control of the expression of *pqsH* in this study. To identify new genes involved in synthesis of PQS, a transposon mutagenesis library was constructed and PA1429 was found to inhibit PQS biosynthesis. We identified that inactivation of PA1429 in *P. aeruginosa* resulted in increased expression of *pqsH* and thus in overproduction of PQS. PA1429 is predicted to encode a probable cation-transporting P-type ATPase. It was found to have an effect on maintenance of calcium homeostasis in *P. aeruginosa* (Guragain et al., 2013). Ca^2+^ has been implicated in various physiological processes such as biofilm formation and virulence factor production (Sarkisova et al., 2005; Patrauchan et al., 2007). Previous studies have shown that PQS signaling regulates numerous virulence factors, motility, and biofilm formation (Reen et al., 2011). In biofilm formation, the *pqs* system is responsible for an increase production of extracellular DNA (Allesen-Holm et al., 2006). In strains defective for PQS, mature biofilm aggregates never fully develop (Passos da Silva et al., 2017). In the present study, our data showed that deletion of PA1429 resulted in increased bacterial motility and biofilm formation (**Figure 3D**). In addition, PQS as an endogenous stress factor predisposes bacteria to developing exogenous stress, the addition of PQS enhanced the susceptibility of *P. aeruginosa* toward H_2_O_2_ (Haussler and Becker, 2008). In agreement with this observation, the PA1429 mutant exhibited greater sensitivity toward H_2_O_2_ than PAO1, while the ΔPA1429Δ*pqsH* double mutant displayed the same levels of sensitivity as the wild-type parent strain (**Figure 4**). This result clearly demonstrated that the PA1429 mutant was more sensitive to H_2_O_2_ due to the production of high levels of PQS.

**FIGURE 6:**
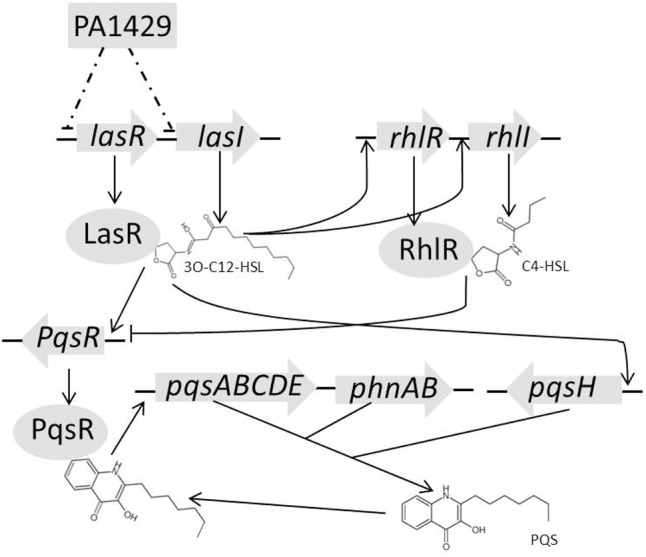
A schematic diagram showing that of PA1429 is involved in the regulation of QS systems in *P. aeruginosa*. The potential regulatory diagram is based on our observations and those of previous studies. Arrows indicate positive regulation and bars indicate negative regulation.

It has been demonstrated that *las, rhl*, and *pqs* system are regulated hierarchically (Lee and Zhang, 2015). LasR regulates RhlR and PQS positively, while RhlR regulates PQS negatively (Kariminik et al., 2017). The transcription of the *pqsABCDE* promoter was positively regulated by LasR and negatively by RhlR indirectly through PqsR (Wade et al., 2005). PqsR is a LysR-type transcriptional regulator that binds to the promoter region of the *pqsABCD* operon and directly controls the expression of the operon. PQS, in turn, was found to be able to enhance the transcription of *rhlI*, thus influencing the RhlR-dependent gene expression. These three systems are arranged hierarchically with the *las* system positively regulating the components of the PQS and *rhl* systems (Lee and Zhang, 2015). Biosynthesis of PQS depends on the *pqsABCDE* operon, *pqsH*, and *pqsR*. Our data showed that the expression of *lasR, rhlI, pqsH*, and *pqsR* were increased in PA1429 mutant compared with that of in PAO1 (**Figure 2**). PA1429 is located upstream of *lasR*. Therefore, we intended to investigate the interaction between PA1429 and *las* signaling system. Our results revealed that PA1429 repressed the expression *lasR* and *lasI*, suggesting that it had a regulatory effect on the *las* system. Previous studies have demonstrated that LasR–3-oxo-C_12_-HSL positively influenced the expression of *rhlR* and *rhlI, pqsH* and *pqsR*; PqsR in turn upregulated *pqsABCDE* (Latifi et al., 1996; Pesci et al., 1997; Whiteley et al., 1999; Wade et al., 2005). Based on our results, we speculated that PA1429 might influence the expression of PQS and *rhl* systems through the *las* system (**Figure 6**). The elevated expression of the QS genes in the PA1429 mutant then activated the downstream virulence factors. Consistent with the QS regulated by PA1429, mutation of PA1429 increases *P. aeruginosa* virulence in a mouse model of acute pneumonia. Overall, these results clearly demonstrated that PA1429 is required for *P. aeruginosa* pathogenesis.

Collectively, our data provide insight into understanding the QS signaling networks of *P. aeruginosa*. Nevertheless, how the PA1429 influences the QS remains unknown. Further work needs to explore other functions of PA1429 and investigate the molecular mechanisms involved in QS and pathogenesis.

## Author Contributions

YZ and HL: conception and design of the study, analysis, and interpretation of data, drafting the manuscript. JQ, BT, WK, CZ, and GC: acquisition of data and analysis of data. All the authors participated the idea discussion and reviewed the manuscript.

## Conflict of Interest Statement

The authors declare that the research was conducted in the absence of any commercial or financial relationships that could be construed as a potential conflict of interest.

## Footnotes

1 http://www.pseudomonas.com
