# Supplementary material for: The P-Type ATPase PA1429 Regulates Quorum-Sensing Systems and Bacterial Virulence
Source: Front Microbiol. 2017 Dec 7;8:2449. doi: 10.3389/fmicb.2017.02449 (PMC5770612; doi:10.3389/fmicb.2017.02449)
Supplement: Supplementary file 1 [file Table_1.docx]

**Table S1.** Bacterial strains, plasmids, and primers

| **Strain, plasmids and primers** | **Related characteristics** | | **Source or reference** |
| --- | --- | --- | --- |
| **Strains** |  | |  |
| ***E. coli*** |  | |  |
| DH5a | *endA hsdR17 supE44 thi-1 recA1 gyrA relA1*Δ(*lacZYA-argF*)*U169 deoR* (*φ80dlac*D(*lacZ*)*M15*) | | Lab stock |
| ***P. aeruginosa*** |  | |  |
| PAO1 | Wild type | | Lab stock |
| ΔPA1429 | PA1429 knockout mutant of PAO1 | | This study |
| Δ*pqsH* | *pqsH* knockout mutant of PAO1;Gm | | ([Zhao et al., 2016](#_ENREF_5)) |
| ΔPA1429Δ*pqsH* | PA1429 and *pqsH* knockout mutant of PAO1; | | This study |
| ΔPA1429/CTX-1429 | Δ*1429* carrying plasmids of mini-CTX-1429*-lux* | | This study |
| **Plasmids** |  | |  |
| pEX18Ap | Gene replacement vector, mob^+^*sacB*, Ap^r^ | | This study |
| pBT20 | Mini-TnA/delivery vector; Gm^r^ | | ([Kulasekara et al., 2005](#_ENREF_3)) |
| pEX18Ap::*1429* | pEX18Ap derivative, for replacing PAO1 *1429* gene with a gentamicin resistance cassette from plasmid pPS858 | | This study |
| pMS402 | *lux-*based promoter reporter plasmid, Km^r^ Tp^r^ | | ([Duan et al., 2003](#_ENREF_1)) |
| *pqsH-lux* | pMS402 containing *pqsH* promoter region | | This study |
| *pqsR-lux* | pMS402 containing p*qsR* promoter region | | This study |
| *pqsA-lux* | pMS402 containing *pqsA* promoter region | | This study |
| *lasI-lux* | pMS402 containing *lasI* promoter region | | ([Liang et al., 2012](#_ENREF_4)) |
| *lasR-lux* | pMS402 containing *lasR* promoter region | | ([Liang et al., 2012](#_ENREF_4)) |
| *rhlI-lux* | pMS402 containing *rhlI* promoter region | | Lab stock |
| *rhlR-lux* | pMS402 containing *rhlR* promoter region | | Lab stock |
| Mini-CTX1 | Gene delivery vector for inserting genes at the CTX phage *att* site on *P. aeruginosa* chromosome | | ([Hoang et al., 2000](#_ENREF_2)) |
| Mini-CTX-1429 | PCR fragment covering the entire PA1429 gene | | This study |
| **Primers** | | | |
| 1429-up-s | | ATAgaattcCCGGCCAGGTAGAAGAAG | |
| 1429- up-a | | AAAggatccGAAGACAGGGGTGGGATG | |
| 1429-down-s | | ATTggatccGGAAAAGTGGCTATGTCG | |
| 1429-down-a | | CTCaagcttAGCGTACAGTCGGAAAAG | |
| 1429-up | | ACTaagcttTTCATCAGAACGAGGCGC | |
| 1429- down | | TCAggatccCACTTTTCCAGTTCGGCG | |

**References**

Duan, K., Dammel, C., Stein, J., Rabin, H., and Surette, M.G. (2003). Modulation of *Pseudomonas aeruginosa* gene expression by host microflora through interspecies communication. *Mol Microbiol* 50(5)**,** 1477-1491.

Hoang, T.T., Kutchma, A.J., Becher, A., and Schweizer, H.P. (2000). Integration-proficient plasmids for *Pseudomonas aeruginosa*: site-specific integration and use for engineering of reporter and expression strains. *Plasmid* 43(1)**,** 59-72. doi: 10.1006/plas.1999.1441.

Kulasekara, H.D., Ventre, I., Kulasekara, B.R., Lazdunski, A., Filloux, A., and Lory, S. (2005). A novel two-component system controls the expression of Pseudomonas aeruginosa fimbrial cup genes. *Mol Microbiol* 55(2)**,** 368-380. doi: 10.1111/j.1365-2958.2004.04402.x.

Liang, H., Deng, X., Ji, Q., Sun, F., Shen, T., and He, C. (2012). The *Pseudomonas aeruginosa* global regulator VqsR directly inhibits QscR to control quorum-sensing and virulence gene expression. *J Bacteriol* 194(12)**,** 3098-3108. doi: 10.1128/JB.06679-11.

Zhao, J., Yu, X., Zhu, M., Kang, H., Ma, J., Wu, M., et al. (2016). Structural and Molecular Mechanism of CdpR Involved in Quorum-Sensing and Bacterial Virulence in *Pseudomonas aeruginosa*. *PLoS Biol* 14(4)**,** e1002449. doi: 10.1371/journal.pbio.1002449.
